# MRM-DIFF: data processing strategy for differential analysis in large scale MRM-based lipidomics studies

**DOI:** 10.3389/fgene.2014.00471

**Published:** 2015-01-30

**Authors:** Hiroshi Tsugawa, Erika Ohta, Yoshihiro Izumi, Atsushi Ogiwara, Daichi Yukihira, Takeshi Bamba, Eiichiro Fukusaki, Masanori Arita

**Affiliations:** ^1^Metabolome Informatics Research Team, Metabolomics Research Group, RIKEN Center for Sustainable Resource ScienceYokohama, Japan; ^2^Department of Biotechnology, Graduate School of Engineering, Osaka UniversitySuita, Osaka, Japan; ^3^Reifycs Inc.Tokyo, Japan; ^4^National Institute of GeneticsShizuoka, Japan

**Keywords:** multiple reaction monitoring, differential analysis, lipidomics, compound identification, isotopic peak estimation

## Abstract

Based on theoretically calculated comprehensive lipid libraries, in lipidomics as many as 1000 multiple reaction monitoring (MRM) transitions can be monitored for each single run. On the other hand, lipid analysis from each MRM chromatogram requires tremendous manual efforts to identify and quantify lipid species. Isotopic peaks differing by up to a few atomic masses further complicate analysis. To accelerate the identification and quantification process we developed novel software, MRM-DIFF, for the differential analysis of large-scale MRM assays. It supports a correlation optimized warping (COW) algorithm to align MRM chromatograms and utilizes quality control (QC) sample datasets to automatically adjust the alignment parameters. Moreover, user-defined reference libraries that include the molecular formula, retention time, and MRM transition can be used to identify target lipids and to correct peak abundances by considering isotopic peaks. Here, we demonstrate the software pipeline and introduce key points for MRM-based lipidomics research to reduce the mis-identification and overestimation of lipid profiles. The MRM-DIFF program, example data set and the tutorials are downloadable at the “Standalone software” section of the PRIMe (Platform for RIKEN Metabolomics, http://prime.psc.riken.jp/) database website.

## Introduction

Multiple reaction monitoring (MRM) during liquid chromatography coupled to a triple quadrupole mass spectrometer (LC/QqQ/MS) is one of the standard methods in lipidomics research (Shaner et al., 2009; Quehenberger et al., 2010). Its highly sensitive and selective performance allows for the reliable monitoring of lipid compounds of low abundance such as oxidized lipids when MRM transitions, i.e., precursor- and product ion pairs, are conditioned appropriately (Uchikata et al., 2012). The key to success in lipidomics are lipid databases such as LIPID MAPS (Fahy et al., 2007) and LipidBlast (Kind et al., 2013) that provide *in silico* MS/MS spectra (and thereby MRM transitions) of major lipid classes such as glycerolipids, phospholipids, and sphingolipids. Thus, large-scale MRM assays monitor 500–1000 “theoretical” transitions on high-end QqQ/MS platforms (Ikeda et al., 2008).

Compared to production scanning by LC quadrupole- time-of-flight or Orbitrap MS (Q-TOF or Q-Orbitrap), the drawback of such theoretical MRM assays is the uncertain reliability of compound identification (Kind et al., 2013; Perez-Riverol et al., 2013). This is largely due to the scan speed of QqQ/MS; it is not fast enough to monitor three or more fragment ions to determine their lipid class and acyl chain properties. The importance of diagnostic transitions has been emphasized in metabolomics (Stein and Heller, 2006; Tsugawa et al., 2013) and it also applies for lipidomics. For example, the notation of phosphatidyl choline (PC) 38:2 [M+H]+ can match more than 100 acyl chain combinations, at least in theory, and they are expected to elute almost simultaneously at the transition 814.6->184.1. Moreover, the exact retention times for these of lipid isomers are currently unavailable.

To solve this problem by informatics we presented a “pattern recognition” approach (Sugimoto et al., 2012) for MRM assays. We called it “differential analysis” for multiple chromatograms because reliable ions and their isotopic ions can be estimated by comparing multiple chromatograms. In addition, candidate annotations can be reduced by statistical analyses before confirming each peak by authentic standards (Sugimoto et al., 2009).

In exchange, to accurately perform MRM based lipidomics with the differential analysis approach, it necessitates the standardized management of all experimental processes from experimental design to data processing. From the experimental design, peak alignment parameters can be determined and signal intensity drifts adjusted. Based on information of isotopic ions, peak- identification can be corrected and quantification can be adjusted because isotopic peaks from abundant ions sometimes overlap with ions of minor lipids. To get around such processes for large datasets we developed a software program that facilitates the differential analysis of large-scale MRM-based lipidomics. Our Multiple Reaction Monitoring-based DIFFerential (MRM-DIFF) analysis software supports chromatographic alignment and compound identification with estimation of isotopic peaks. Here we introduce a systematic strategy to perform differential analysis by MRM-DIFF with pooled quality control (QC) datasets. The pooled QC data, a mixture of small aliquots from each sample, were originally used to correct MS intensity drifts across a given analytical batch (Dunn et al., 2011). We use them to select a suitable “reference” for chromatographic alignment and peak quantification and demonstrate the advantages of our strategy with 37 serum datasets with 189 MRM transitions each.

## Results

**Figure 1:**
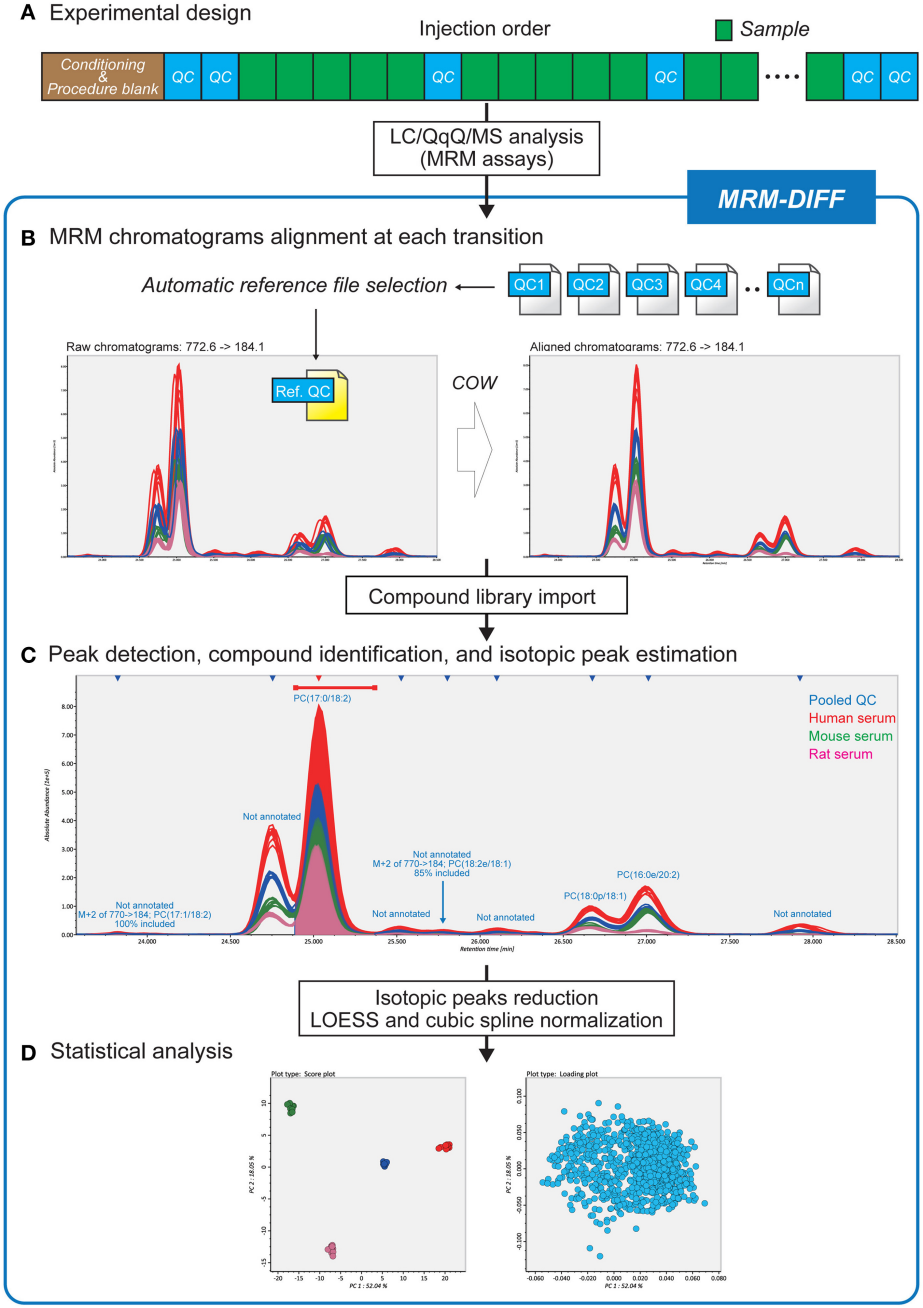
**Differential analysis strategy for large-scale MRM-based lipidomics**. **(A)** Since this strategy utilizes pooled quality control (QC) datasets for data processing methods, pooled QCs are injected at every five biological samples. **(B)** After reference file selection based on chromatograms of pooled QC datasets, MRM chromatograms are adjusted to the MRM chromatogram reference file by correlation optimized warping based non-linear alignment. **(C)** The data processing results including compound identifications and isotopic peak estimations can be monitored in the MRM-DIFF program. The graphical user interface facilitates manual curation of the results as well as validation of identification accuracy. **(D)** In addition to principal component analysis, MRM-DIFF supports standardization methods including isotopic peak reduction and LOESS/cubic spline based normalization.

Figure 1 is a summary of our data processing method. After LC conditioning, pooled QC samples are analyzed at each fifth sample injection (Figure 1A). Based on the “chromatographic centroid” concept (see **Theory** below), one QC dataset is automatically selected from among all raw data files to serve as the reference. All other chromatograms, including the QC datasets, are aligned to the selected reference chromatogram by correlation optimized warping (COW), a popular dynamic programming algorithm for non-linear chromatographic alignment (Figure 1B) (Nielsen et al., 1998; Tomasi et al., 2004). Then a user-defined library of the retention time, the MRM transition, and the molecular formula for compounds is imported to perform peak identification and isotopic estimation. The program also implements a peak detection algorithm and uses the abundance of detected peaks for multivariate analysis (Figure 1C). The QC data are also exploited in the peak detection process. Since the pooled QC data are expected to contain all detectable lipid compounds from all samples, missing peaks, i.e., existing but unidentified peaks in some biological samples, can be interpolated based on the peak widths and retention times of the QC peaks. The algorithmic detail is described in the **Theory** section. The graphic user interface assists the workflow and contributes to a better understanding of compound identification and peak quantification (Figure 1C). The abundances of isotopic peaks are resolved and signal intensity drifts are corrected with LOESS (Cleveland, 1979) and cubic spline before statistical analyses (Figure 1D).

In our proof-of-concept analysis, MRM-DIFF successfully profiled 259 lipids and 677 unknown metabolites in human-, mouse-, and rat-serum using 189 MRM transitions (see Supplementary File 1 for all profiles). Details of the MRM transitions and retention times of our focused lipids are shown in Table 1. The time for data processing was less than 20 min (Intel Core i7-4700MQ CPU at 2.4 Gb and 8 Gb RAM with Windows 8.1). For a few un-annotated peaks, the identification results were manually curated through the graphical user interface (GUI) of the MRM-DIFF program. The lipid coverage for the PC and phosphatidylethanolamine (PE) species on LC/QqQ/MS exceeded previously reported results (Psychogios et al., 2011).

**Table 1:** **Detailed MRM conditions and retention times of targeted lipids**.

**Name**	**Formula**	**Precursor *m/z***	**Product *m/z***	**Retention time [min]**	**Ion mode**	**Event ID**	**Dwell time [ms]**	**Q1 Pre bias [V]**	**Collision energy [V]**	**Q3 Pre bias [V]**
lysoPC 14:0 (*sn*-2)	C22H46NO7P	468.3	184.1	6.34	Positive	1	5	20	20	25
lysoPC 14:0 (*sn*-1)	C22H46NO7P	468.3	184.1	7.03	Positive	1	5	20	20	25
lysoPC 15:0 (*sn*-2)	C23H48NO7P	482.3	184.1	7.88	Positive	2	5	20	20	25
lysoPC 15:0 (*sn*-1)	C23H48NO7P	482.3	184.1	8.65	Positive	2	5	20	20	25
lysoPC 16:0e	C24H52NO6P	482.3	184.1	12.20	Positive	2	5	20	20	25
lysoPC 15:1 (*sn*-1)	C23H48NO7P	480.3	184.1	7.23	Positive	3	5	20	20	25
lysoPC 16:0p	C24H50NO6P	480.3	184.1	11.86	Positive	3	5	20	20	25
lysoPC 16:0 (*sn*-2)	C23H46NO7P	496.3	184.1	9.58	Positive	4	5	20	20	25
lysoPC 16:0 (*sn*-1)	C24H50NO7P	496.3	184.1	10.40	Positive	4	5	20	20	25
lysoPC 16:1 (*sn*-2)	C24H48NO7P	494.3	184.1	7.29	Positive	5	5	20	20	25
lysoPC 16:1 (*sn*-1)	C24H48NO7P	494.3	184.1	8.00	Positive	5	5	20	20	25
lysoPC 17:0 (*sn*-2)	C25H52NO7P	510.4	184.1	11.29	Positive	6	5	20	20	25
lysoPC 17:0 (*sn*-1)	C25H52NO7P	510.4	184.1	12.10	Positive	6	5	20	20	25
lysoPC 17:1 (*sn*-2)	C25H50NO7P	508.3	184.1	8.89	Positive	7	5	20	20	25
lysoPC 17:1 (*sn*-1)	C25H50NO7P	508.3	184.1	9.62	Positive	7	5	20	20	25
lysoPC 18:0p	C26H54NO6P	508.3	184.1	15.07	Positive	7	5	20	20	25
lysoPC 18:0 (*sn*-2)	C26H54NO7P	524.4	184.1	12.93	Positive	8	5	20	20	25
lysoPC 18:0 (*sn*-1)	C26H54NO7P	524.4	184.1	13.72	Positive	9	5	20	20	25
lysoPC 18:1 (*sn*-2)	C26H52NO7P	522.4	184.1	10.51	Positive	10	5	20	20	25
lysoPC 18:1 (*sn*-1)	C26H52NO7P	522.4	184.1	11.27	Positive	10	5	20	20	25
lysoPC 18:2 (*sn*-2)	C26H50NO7P	520.3	184.1	8.53	Positive	11	5	20	20	25
lysoPC 18:2 (*sn*-1)	C26H50NO7P	520.3	184.1	9.27	Positive	11	5	20	20	25
lysoPC 18:3 (*sn*-2)	C26H48NO7P	518.3	184.1	6.95	Positive	12	5	20	20	25
lysoPC 18:3 (*sn*-1)	C26H48NO7P	518.3	184.1	7.66	Positive	12	5	20	20	25
lysoPC 19:0 (*sn*-2)	C27H56NO7P	538.4	184.1	14.51	Positive	13	5	20	20	25
lysoPC 19:0 (*sn*-1)	C27H56NO7P	538.4	184.1	15.27	Positive	13	5	20	20	25
lysoPC 20:0 (*sn*-2)	C28H58NO7P	552.4	184.1	15.98	Positive	14	5	20	20	25
lysoPC 20:0 (*sn*-1)	C28H58NO7P	552.4	184.1	16.67	Positive	14	5	20	20	25
lysoPC 20:1 (*sn*-2)	C28H56NO7P	550.4	184.1	13.58	Positive	15	5	20	20	25
lysoPC 20:1 (*sn*-1)	C28H56NO7P	550.4	184.1	14.36	Positive	15	5	20	20	25
lysoPC 20:2 (*sn*-2)	C28H54NO7P	548.4	184.1	11.60	Positive	16	5	20	20	25
lysoPC 20:2 (*sn*-1)	C28H54NO7P	548.4	184.1	12.38	Positive	16	5	20	20	25
lysoPC 20:3 (*sn*-2)	C28H52NO7P	546.4	184.1	9.86	Positive	17	5	20	20	25
lysoPC 20:3 (*sn*-1)	C28H52NO7P	546.4	184.1	10.57	Positive	17	5	20	20	25
lysoPC 20:4 (*sn*-2)	C28H50NO7P	544.3	184.1	8.63	Positive	18	5	20	20	25
lysoPC 20:4 (*sn*-1)	C28H50NO7P	544.3	184.1	9.29	Positive	18	5	20	20	25
lysoPC 20:5 (*sn*-2)	C28H48NO7P	542.3	184.1	7.09	Positive	19	5	20	20	25
lysoPC 20:5 (*sn*-1)	C28H48NO7P	542.3	184.1	7.70	Positive	19	5	20	20	25
lysoPC 22:0 (*sn*-2)	C30H62NO7P	580.4	184.1	18.53	Positive	20	5	20	20	25
lysoPC 22:0 (*sn*-1)	C30H62NO7P	580.4	184.1	19.17	Positive	20	5	20	20	25
lysoPC 22:4 (*sn*-2)	C30H54NO7P	572.4	184.1	11.23	Positive	21	5	20	20	25
lysoPC 22:4 (*sn*-1)	C30H54NO7P	572.4	184.1	11.92	Positive	21	5	20	20	25
lysoPC 22:6 (*sn*-2)	C30H50NO7P	568.3	184.1	8.69	Positive	22	5	20	20	25
lysoPC 22:6 (*sn*-1)	C30H50NO7P	568.3	184.1	9.33	Positive	22	5	20	20	25
PC 24:0; PC 12:0/12:0 (IS)	C32H64NO8P	622.4	184.1	17.70	Positive	23	5	24	26	26
PC 30:0; PC 14:0/16:0	C38H76NO8P	706.5	184.1	23.39	Positive	24	5	24	26	26
PC 30:1; PC 14:0/16:1	C38H74NO8P	704.5	184.1	22.16	Positive	25	5	24	26	26
PC 31:0; PC 15:0/16:0	C39H78NO8P	720.5	184.1	24.16	Positive	26	5	24	26	26
PC 32:0e; PC 16:0e/16:0	C39H80NO7P	720.5	184.1	26.02	Positive	26	5	24	26	26
PC 31:1; PC 15:0/16:1	C39H76NO8P	718.5	184.1	22.97	Positive	27	5	24	26	26
PC 32:0p; PC 16:0p/16:0	C39H76NO7P	718.5	184.1	25.72	Positive	27	5	24	26	26
PC 32:0; PC 16:0/16:0	C40H80NO8P	734.6	184.1	24.97	Positive	28	5	24	26	26
PC 32:1; PC 14:0/18:1 or 16:0/16:1	C40H78NO8P	732.5	184.1	23.74	Positive	29	5	24	26	26
PC 32:2; PC 14:0/18:2 or 16:1/16:1	C40H76NO8P	730.5	184.1	22.69	Positive	30	5	24	26	26
PC 33:0; PC 17:0/16:0 or 18:0/15:0	C41H82NO8P	748.6	184.1	25.74	Positive	31	5	24	26	26
PC 34:0e; PC 18:0e/16:0	C41H84NO7P	748.6	184.1	27.88	Positive	31	5	24	26	26
PC 33:1; PC 15:0/18:1 or 16:0/17:1	C41H80NO8P	746.6	184.1	24.50	Positive	32	5	24	26	26
PC 34:1e; PC 18:0e/16:1 or 18:1e/16:0	C41H82NO7P	746.6	184.1	26.32	Positive	32	5	24	26	26
PC 33:2; PC 15:0/18:2	C41H78NO8P	744.6	184.1	23.47	Positive	33	5	24	26	26
PC 34:2e; PC 16:0e/18:2	C41H80NO7P	744.6	184.1	24.97	Positive	33	5	24	26	26
PC 34:2e; PC 16:1e/18:1	C41H80NO7P	744.6	184.1	25.23	Positive	33	5	24	26	26
PC 34:0; PC 16:0/18:0	C42H84NO8P	762.6	184.1	26.67	Positive	34	5	24	26	26
PC 34:1; PC 16:0/18:1	C42H82NO8P	760.6	184.1	25.27	Positive	35	5	24	26	26
PC 34:2; PC 16:0/18:2 or 16:1/18:1	C42H80NO8P	758.6	184.1	24.22	Positive	36	5	24	26	26
PC 34:3; PC 14:0/20:3	C42H78NO8P	756.6	184.1	23.05	Positive	37	5	24	26	26
PC 34:3; PC 16:0/18:3 or 16:1/18:2	C42H78NO8P	756.6	184.1	23.41	Positive	37	5	24	26	26
PC 34:4; PC 14:0/20:4 or 16:1/18:3	C42H76NO8P	754.5	184.1	22.62	Positive	38	5	24	26	26
PC 34:5; PC 14:0/20:5	C42H74NO8P	752.5	184.1	21.74	Positive	39	5	24	26	26
PC 35:1; PC 16:0/19:1 or 17:0/18:1 or 17:1/18:0	C43H84NO8P	774.6	184.1	26.06	Positive	40	5	24	26	26
PC 36:2e; PC 16:0e/20:2	C43H84NO7P	772.6	184.1	27.01	Positive	41	5	24	26	26
PC 35:2; PC 17:0/18:2 or 17:1/18:1	C43H82NO8P	772.6	184.1	25.03	Positive	41	5	24	26	26
PC 35:2e; PC 18:0p/18:1 or 18:1e/18:1	C43H84NO7P	772.6	184.1	26.67	Positive	41	5	24	26	26
PC 35:3; PC 17:1/18:2	C43H80NO8P	770.6	184.1	23.82	Positive	42	5	24	26	26
PC 36:3e; PC 18:1e/18:2	C43H82NO7P	770.6	184.1	25.55	Positive	42	5	24	26	26
PC 36:3e; PC 18:2e/18:1	C43H82NO7P	770.6	184.1	25.80	Positive	42	5	24	26	26
PC 35:4; PC 15:0/20:4	C43H78NO8P	768.6	184.1	23.37	Positive	43	5	24	26	26
PC 36:4e; PC 16:1e/20:3	C43H78NO8P	768.6	184.1	25.11	Positive	43	5	24	26	26
PC 35:5; PC 15:0/20:5	C43H76NO8P	766.5	184.1	22.54	Positive	44	5	24	26	26
PC 36:5e; PC 16:0e/20:5	C43H78NO7P	766.5	184.1	24.30	Positive	44	5	24	26	26
PC 36:4p; PC 16:0p/20:4	C43H76NO7P	766.5	184.1	24.83	Positive	44	5	24	26	26
PC 38:6p; PC 16:0p/22:6	C44H88NO7P	790.6	184.1	29.41	Positive	45	5	24	26	26
PC 36:0; PC 18:0/18:0	C44H88NO8P	790.6	184.1	28.66	Positive	45	5	24	26	26
PC 36:1; PC 18:0/18:1	C44H86NO8P	788.6	184.1	27.07	Positive	46	5	24	26	26
PC 36:2; PC 18:0/18:2 or 18:1/18:1	C44H84NO8P	786.6	184.1	25.82	Positive	47	5	24	26	26
PC 36:3; PC 16:0/20:3 or 18:0/18:3 or 18:1/18:2	C44H82NO8P	784.6	184.1	24.66	Positive	48	5	24	26	26
PC 36:4; PC 16:0/20:4 or 16:1/20:3	C44H80NO8P	782.6	184.1	24.14	Positive	49	5	24	26	26
PC 36:4; PC 18:1/18:3 or 18:2/18:2	C44H80NO8P	782.6	184.1	23.55	Positive	49	5	24	26	26
PC 36:5; PC 14:0/22:5 or 16:0/20:5 or 16:1/20:4	C44H78NO8P	780.6	184.1	23.29	Positive	50	5	24	26	26
PC 36:5; PC 18:2/18:3	C44H78NO8P	780.6	184.1	22.93	Positive	50	5	24	26	26
PC 36:6; PC 14:0/22:6	C44H76NO8P	778.5	184.1	22.48	Positive	51	5	24	26	26
PC 36:6; PC 16:1/20:5	C44H76NO8P	778.5	184.1	22.14	Positive	51	5	24	26	26
PC 37:1; PC 18:0/19:1 or 19:0/18:1	C45H88NO8P	802.6	184.1	27.96	Positive	52	5	24	26	26
PC 37:2; PC 18:1/19:1 or 18:2/19:0	C45H86NO8P	800.6	184.1	26.70	Positive	53	5	24	26	26
PC 37:3; PC 17:0/20:3 or 19:1/18:2	C45H84NO8P	798.6	184.1	25.59	Positive	54	5	24	26	26
PC 37:4; PC 17:0/20:4	C45H82NO8P	796.6	184.1	24.91	Positive	55	5	24	26	26
PC 38:4e; PC 18:0e/20:4	C45H84NO7P	796.6	184.1	26.85	Positive	55	5	24	26	26
PC 38:4e; PC 18:1e/20:3	C45H84NO7P	796.6	184.1	26.40	Positive	55	5	24	26	26
PC 38:5e; PC 16:0e/22:5 or 18:0e/20:5	C45H82NO7P	794.6	184.1	25.41	Positive	56	5	24	26	26
PC 37:5; PC 17:0/20:5 or 17:1/20:4	C45H80NO8P	794.6	184.1	24.10	Positive	56	5	24	26	26
PC 38:4p; PC 18:0p/20:4	C45H80NO7P	794.6	184.1	26.08	Positive	56	5	24	26	26
PC 37:6; PC 15:0/22:6	C45H78NO8P	792.6	184.1	23.29	Positive	57	5	24	26	26
PC 38:6e; PC 16:0e/22:6	C45H80NO7P	792.6	184.1	25.13	Positive	57	5	24	26	26
PC 38:6e; PC 18:1e/20:5	C45H80NO7P	792.6	184.1	24.38	Positive	57	5	24	26	26
PC 38:1; PC 18:1/20:0	C46H90NO8P	816.6	184.1	29.01	Positive	58	5	24	26	26
PC 38:2; PC 16:0/22:2	C46H88NO8P	814.6	184.1	27.22	Positive	59	5	24	26	26
PC 38:2; PC 18:0/20:2	C46H88NO8P	814.6	184.1	27.49	Positive	59	5	24	26	26
PC 38:3; PC 18:0/20:3 or 18:1/20:2	C46H86NO8P	812.6	184.1	26.44	Positive	60	5	24	26	26
PC 38:4; PC 18:0/20:4	C46H84NO8P	810.6	184.1	25.73	Positive	61	5	24	26	26
PC 38:4; PC 18:1/20:3	C46H84NO8P	810.6	184.1	25.37	Positive	61	5	24	26	26
PC 38:5; PC 18:0/20:5	C46H82NO8P	808.6	184.1	24.86	Positive	62	5	24	26	26
PC 38:5; PC 18:1/20:4	C46H82NO8P	808.6	184.1	24.46	Positive	62	5	24	26	26
PC 38:6; PC 16:0/22:6 or 18:2/20:4	C46H80NO8P	806.6	184.1	24.02	Positive	63	5	24	26	26
PC 38:7; PC 16:1/22:6 or 18:2/20:5	C46H78NO8P	804.6	184.1	22.62	Positive	64	5	24	26	26
PC 39:3; PC 19:0/20:3	C47H88NO8P	826.6	184.1	27.33	Positive	65	5	24	26	26
PC 39:6; PC 17:0/22:6	C47H84NO8P	820.6	184.1	24.76	Positive	66	5	24	26	26
PC 40:5p; PC 18:0p/22:5	C47H84NO7P	820.6	184.1	26.60	Positive	66	5	24	26	26
PC 40:6e; PC 18:1e/22:5	C47H86NO7P	820.6	184.1	25.71	Positive	66	5	24	26	26
PC 39:7; PC 17:1/22:6	C47H80NO8P	818.6	184.1	24.18	Positive	67	5	24	26	26
PC 40:7e; PC 18:1e/22:6	C47H82NO7P	818.6	184.1	25.31	Positive	67	5	24	26	26
PC 40:1; PC 18:1/22:0	C48H94NO8P	844.7	184.1	31.51	Positive	68	5	24	26	26
PC 40:4; PC 18:0/22:4 or 20:1/20:3	C48H88NO8P	838.6	184.1	27.57	Positive	69	5	24	26	26
PC 40:5; PC 18:0/22:5	C48H86NO8P	836.6	184.1	26.54	Positive	69	5	24	26	26
PC 40:6; PC 18:0/22:6	C48H84NO8P	834.6	184.1	25.59	Positive	70	5	24	26	26
PC 40:6; PC 18:1/22:5 or 20:2/20:4	C48H84NO8P	834.6	184.1	25.13	Positive	70	5	24	26	26
PC 40:7; PC 18:1/22:6	C48H82NO8P	832.6	184.1	24.28	Positive	71	5	24	26	26
PC 40:7; PC 20:3/20:4	C48H82NO8P	832.6	184.1	23.91	Positive	71	5	24	26	26
PC 40:8; PC 20:4/20:4	C48H80NO8P	830.6	184.1	23.27	Positive	72	5	24	26	26
PC 41:6; PC 19:0/22:6	C49H86NO8P	848.6	184.1	26.42	Positive	73	5	24	26	26
lysoPE 14:0 (*sn*-2)	C19H40NO7P	426.3	285.3	6.40	Positive	74	5	17	15	25
lysoPE 14:0 (*sn*-1)	C19H40NO7P	426.3	285.3	7.25	Positive	74	5	17	15	25
lysoPE 16:0 (*sn*-2)	C21H44NO7P	454.3	313.3	9.71	Positive	75	5	17	15	25
lysoPE 16:0 (*sn*-1)	C21H44NO7P	454.3	313.3	10.52	Positive	75	5	17	15	25
lysoPE 17:0 (*sn*-2)	C22H46NO7P	468.3	327.3	11.45	Positive	76	5	17	15	25
lysoPE 17:0 (*sn*-1)	C22H46NO7P	468.3	327.3	12.22	Positive	76	5	17	15	25
lysoPE 18:0e	C23H50NO6P	468.3	327.3	15.47	Positive	76	5	17	15	25
lysoPE 18:0 (*sn*-2)	C23H48NO7P	482.3	341.3	13.06	Positive	77	5	17	15	25
lysoPE 18:0 (*sn*-1)	C23H48NO7P	482.3	341.3	13.83	Positive	77	5	17	15	25
lysoPE 18:1 (*sn*-2)	C23H46NO7P	480.3	339.3	10.66	Positive	78	5	17	15	25
lysoPE 18:1 (*sn*-1)	C23H46NO7P	480.3	339.3	11.35	Positive	78	5	17	15	25
lysoPE 18:2 (*sn*-2)	C23H44NO7P	478.3	337.3	8.62	Positive	79	5	17	15	25
lysoPE 18:2 (*sn*-1)	C23H44NO7P	478.3	337.3	9.37	Positive	79	5	17	15	25
lysoPE 20:0 (*sn*-2)	C25H52NO7P	510.4	369.4	15.99	Positive	80	5	17	15	25
lysoPE 20:0 (*sn*-1)	C25H52NO7P	510.4	369.4	16.74	Positive	80	5	17	15	25
lysoPE 20:1 (*sn*-2)	C25H50NO7P	508.3	367.3	13.75	Positive	81	5	17	15	25
lysoPE 20:1 (*sn*-1)	C25H50NO7P	508.3	367.3	14.42	Positive	81	5	17	15	25
lysoPE 20:3 (*sn*-2)	C25H46NO7P	504.3	363.3	10.07	Positive	82	5	17	15	25
lysoPE 20:3 (*sn*-1)	C25H46NO7P	504.3	363.3	10.70	Positive	82	5	17	15	25
lysoPE 20:4 (*sn*-2)	C25H44NO7P	502.3	361.3	8.70	Positive	83	5	17	15	25
lysoPE 20:4 (*sn*-1)	C25H44NO7P	502.3	361.3	9.35	Positive	83	5	17	15	25
lysoPE 20:5 (*sn*-1)	C25H42NO7P	500.3	359.3	7.10	Positive	84	5	17	15	25
lysoPE 20:5 (*sn*-2)	C25H42NO7P	500.3	359.3	7.77	Positive	84	5	17	15	25
lysoPE 22:6 (*sn*-1)	C27H44NO7P	526.3	385.3	8.88	Positive	85	5	17	15	25
lysoPE 22:6 (*sn*-2)	C27H44NO7P	526.3	385.3	9.51	Positive	85	5	17	15	25
PE 32:0; PE 16:0/16:0	C37H74NO8P	692.5	551.5	24.94	Positive	86	5	20	20	30
PE 32:1; PE 14:0/18:1 or 16:0/16:1	C37H72NO8P	690.5	549.5	23.73	Positive	87	5	20	20	30
PE 32:2; PE 14:0/18:2	C37H70NO8P	688.5	547.5	22.60	Positive	88	5	20	20	30
PE 33:1; PE 15:0/18:1	C38H74NO8P	704.6	563.6	24.50	Positive	89	5	20	20	30
PE 34:1e; PE 16:0e/18:1	C38H76NO7P	704.6	563.6	26.32	Positive	89	5	20	20	30
PE 33:2; PE 15:0/18:2	C38H72NO8P	702.5	561.5	23.27	Positive	90	5	20	20	30
PE 34:1p; PE 16:0p/18:1	C38H72NO7P	702.5	561.5	25.23	Positive	90	5	20	20	30
PE 34:0; PE 16:0/18:0	C39H78NO8P	720.6	579.6	26.56	Positive	91	5	20	20	30
PE 34:1; PE 16:0/18:1	C39H76NO8P	718.5	577.5	25.21	Positive	92	5	20	20	30
PE 34:2; PE 16:0/18:2 or 16:1/18:1	C39H74NO8P	716.5	575.5	24.26	Positive	93	5	20	20	30
PE 34:3; PE 16:0/18:3	C39H72NO8P	714.5	573.5	23.00	Positive	94	5	20	20	30
PE 34:3; PE 16:1/18:2	C39H72NO8P	714.5	573.5	23.43	Positive	94	5	20	20	30
PE 35:1; PE 17:0/18:1	C40H78NO8P	732.6	591.6	26.05	Positive	95	5	20	20	30
PE 36:1e; PE 18:0e/18:1	C40H80NO7P	732.6	591.6	28.22	Positive	95	5	20	20	30
PE 35:2; PE 17:0/18:2	C40H76NO8P	730.5	589.5	25.04	Positive	96	5	20	20	30
PE 36:2e; PE 18:0e/18:2	C40H78NO7P	730.5	589.5	27.04	Positive	96	5	20	20	30
PE 36:1p; PE 18:0p/18:1	C40H76NO7P	730.5	589.5	27.97	Positive	96	5	20	20	30
PE 35:3; PE 17:1/18:2	C40H74NO8P	728.5	587.5	24.00	Positive	97	5	20	20	30
PE 36:2p or 36:3e; PE 18:0p/18:2 or 18:0e/18:3 or 18:1p/18:1	C40H74NO7P	728.5	587.5	26.66	Positive	97	5	20	20	30
PE 36:3p or 36:4e; PE 16:0p/20:3 or 16:0e/20:4	C40H72NO7P	726.5	585.5	25.14	Positive	98	5	20	20	30
PE 36:4p; PE 16:0p/20:4	C40H70NO7P	724.5	583.5	24.80	Positive	99	5	20	20	30
PE 36:5p; PE 16:0p/20:5	C40H68NO7P	722.5	581.5	23.70	Positive	100	5	20	20	30
PE 36:1; PE 16:0/20:1 or 18:0/18:1	C41H80NO8P	746.6	605.6	27.00	Positive	101	5	20	20	30
PE 36:2; PE 18:0/18:2 or 18:1/18:1	C41H78NO8P	744.6	603.6	25.79	Positive	102	5	20	20	30
PE 36:3; PE 18:1/18:2	C41H76NO8P	742.5	601.5	24.70	Positive	103	5	20	20	30
PE 36:4; PE 16:0/20:4	C41H74NO8P	740.5	599.5	24.15	Positive	104	5	20	20	30
PE 36:4; PE 18:2/18:2	C41H74NO8P	740.5	599.5	23.55	Positive	104	5	20	20	30
PE 36:5; PE 16:0/20:5	C41H72NO8P	738.5	597.5	23.33	Positive	105	5	20	20	30
PE 36:5; PE 18:2/18:3	C41H72NO8P	738.5	597.5	23.00	Positive	105	5	20	20	30
PE 36:6; PE 16:1/20:5	C41H70NO8P	736.5	595.5	22.00	Positive	106	5	20	20	30
PE 37:2; PE 19:0/18:2	C42H80NO8P	758.6	617.6	26.60	Positive	107	5	20	20	30
PE 38:1e; PE 20:0e/18:1	C42H82NO7P	758.6	617.6	29.04	Positive	107	5	20	20	30
PE 37:3; PE 17:0/20:3	C42H78NO8P	756.6	615.6	25.50	Positive	108	5	20	20	30
PE 37:4; PE 17:0/20:4	C42H76NO8P	754.5	613.5	24.94	Positive	109	5	20	20	30
PE 38:4e; PE 18:0e/20:4 or 20:0e/18:4 or 20:1e/18:3	C42H78NO7P	754.5	613.5	26.88	Positive	109	5	20	20	30
PE 38:4e; PE 18:1e/20:3	C42H78NO7P	754.5	613.5	26.38	Positive	109	5	20	20	30
PE 38:4p; PE 16:0p/22:4 or 18:1p/20:3	C42H74NO7P	752.5	611.5	25.45	Positive	110	5	20	20	30
PE 37:5; PE 17:1/20:4	C42H74NO8P	752.5	611.5	23.30	Positive	110	5	20	20	30
PE 38:4p; PE 18:0p/20:4	C42H74NO7P	752.5	611.5	26.48	Positive	110	5	20	20	30
PE 38:5p or 38:6e; PE 18:0p/20:5 or 18:1p/20:4 or 16:0e/22:6	C42H72NO7P	750.5	609.5	24.92	Positive	111	5	20	20	30
PE 38:6p; PE 16:0p/22:6	C42H70NO7P	748.6	607.6	24.64	Positive	112	5	20	20	30
PE 38:1; PE 18:0/20:1	C43H84NO8P	774.6	633.6	28.94	Positive	113	5	20	20	30
PE 38:2; PE 18:1/20:1	C43H82NO8P	772.6	631.6	27.38	Positive	114	5	20	20	30
PE 38:2; PE 20:0/18:2	C43H82NO8P	772.6	631.6	27.49	Positive	114	5	20	20	30
PE 38:3; PE 18:0/20:3	C43H80NO8P	770.6	629.6	26.39	Positive	115	5	20	20	30
PE 38:4; PE 16:0/22:4	C43H78NO8P	768.6	627.6	25.06	Positive	116	5	20	20	30
PE 38:4; PE 18:0/20:4	C43H78NO8P	768.6	627.6	25.69	Positive	116	5	20	20	30
PE 38:4; PE 18:1/20:3 or 18:2/20:2	C43H78NO8P	768.6	627.6	25.32	Positive	116	5	20	20	30
PE 38:5; PE 18:0/20:5	C43H76NO8P	766.5	625.5	24.86	Positive	117	5	20	20	30
PE 38:5; PE 18:1/20:4	C43H76NO8P	766.5	625.5	24.47	Positive	117	5	20	20	30
PE 38:6; PE 16:0/22:6 or 16:1/22:5 or 20:2/18:4	C43H74NO8P	764.5	623.5	23.97	Positive	118	5	20	20	30
PE 38:6; PE 18:1/20:5 or 18:2/20:4	C43H74NO8P	764.5	623.5	23.44	Positive	118	5	20	20	30
PE 38:7; PE 16:1/22:6 or 18:2/20:5	C43H72NO8P	762.5	621.5	22.40	Positive	119	5	20	20	30
PE 40:4e; PE 18:0e/22:4 or 20:0e/20:4	C44H82NO7P	782.6	641.6	28.86	Positive	120	5	20	20	30
PE 39:4; PE 19:0/20:4	C44H80NO8P	782.6	641.6	26.11	Positive	120	5	20	20	30
PE 39:5; PE 17:0/22:5	C44H78NO8P	780.6	639.6	25.06	Positive	121	5	20	20	30
PE 39:6; PE 17:0/22:6	C44H76NO8P	778.5	637.5	24.50	Positive	122	5	20	20	30
PE 40:5p; PE 18:0p/22:5 or 18:1p/22:4	C44H76NO7P	778.5	637.5	27.36	Positive	122	5	20	20	30
PE 39:7; PE 17:1/22:6	C44H74NO8P	776.5	635.5	23.36	Positive	123	5	20	20	30
PE 40:6p; PE 18:0p/22:6 or 18:1p/22:5	C44H74NO7P	776.5	635.5	26.23	Positive	123	5	20	20	30
PE 40:1; PE 22:0/18:1	C45H88NO8P	802.6	661.6	30.20	Positive	124	5	20	20	30
PE 40:2; PE 18:1/22:1	C45H86NO8P	800.6	659.6	29.00	Positive	125	5	20	20	30
PE 40:2; PE 22:0/18:2	C45H86NO8P	800.6	659.6	29.10	Positive	125	5	20	20	30
PE 40:3; PE 18:1/22:2 or 22:1/18:2	C45H84NO8P	798.6	657.6	28.00	Positive	126	5	20	20	30
PE 40:4; PE 18:0/22:4 or 20:0/20:4	C45H82NO8P	796.6	655.6	27.02	Positive	127	5	20	20	30
PE 40:5; PE 18:0/22:5	C45H80NO8P	794.6	653.6	26.03	Positive	128	5	20	20	30
PE 40:6; PE 18:0/22:6	C45H78NO8P	792.6	651.6	25.54	Positive	129	5	20	20	30
PE 40:6; PE 18:1/22:5	C45H78NO8P	792.6	651.6	25.15	Positive	129	5	20	20	30
PE 40:7; PE 18:1/22:6	C45H76NO8P	790.5	649.5	24.35	Positive	130	5	20	20	30
PE 42:5; PE 22:1/20:4	C47H84NO8P	822.6	681.6	28.00	Positive	131	5	20	20	30
PE 42:7; PE 20:1/22:6	C47H80NO8P	818.6	677.6	26.00	Positive	132	5	20	20	30
PE 42:8; PE 20:2/22:6	C47H78NO8P	816.6	675.6	25.00	Positive	133	5	20	20	30
4-Cholesten-3-one	C27H44O	385.3	108.8	23.26	Positive	134	5	19	10	19
5α-Cholestan-3-one	C27H46O	387.2	94.9	25.60	Positive	135	5	24	10	24
Acylcarnitine 2:0	C9H17NO4	204.1	85.05	0.98	Positive	136	5	10	21	16
Acylcarnitine 4:0	C11H22NO4	233.2	85.05	0.98	Positive	137	5	12	21	16
Acylcarnitine 6:0	C13H26NO4	261.2	85.05	1.20	Positive	138	5	14	22	16
Acylcarnitine 8:0	C15H30NO4	289.2	85.05	1.50	Positive	139	5	15	22	16
Acylcarnitine 10:0	C17H34NO4	317.3	85.05	2.21	Positive	140	5	16	22	16
Acylcarnitine 12:0	C19H38NO4	345.3	85.05	3.72	Positive	141	5	17	23	16
Acylcarnitine 14:0	C21H42NO4	373.3	85.05	6.37	Positive	142	5	18	26	16
Acylcarnitine 14:1	C21H40NO4	371.3	85.05	4.59	Positive	143	5	18	26	16
Acylcarnitine 16:0	C23H46NO4	401.4	85.05	9.62	Positive	144	5	19	27	16
Acylcarnitine 16:1	C23H44NO4	399.3	85.05	7.40	Positive	145	5	19	27	16
Acylcarnitine 18:0	C25H50NO4	429.4	85.05	13.04	Positive	146	5	21	28	16
Acylcarnitine 18:1	C25H48NO4	427.4	85.05	10.59	Positive	147	5	21	28	16
Acylcarnitine 18:2	C25H46NO4	425.4	85.05	8.57	Positive	148	5	21	28	16
FFA 12:0 (Lauric acid)	C12H24O2	199	199	4.53	Negative	149	5	25	10	25
FFA 13:0 (Tridecanoic acid)	C13H26O2	213.2	213.2	5.50	Negative	150	5	17	10	20
FFA 14:0 (Myristic acid)	C14H28O2	227.1	227.1	7.52	Negative	151	5	15	10	15
FFA 14:1 (Myristoleic acid, *n*–5)	C14H26O2	225.2	225.2	5.44	Negative	152	5	15	10	15
FFA 15:0 (Pentadecylic acid)	C15H30O2	240.8	240.8	9.10	Negative	153	5	16	10	16
FFA 16:0 (Palmitic acid)	C16H32O2	255.05	255.05	11.02	Negative	154	5	17	10	17
FFA 16:1 (Palmitoleic acid, *n*–7)	C16H30O2	253.1	253.1	10.59	Negative	155	5	15	10	15
FFA 17:0 (Margaric acid)	C17H34O2	268.9	268.9	12.75	Negative	156	5	13	10	13
FFA 17:1 (*cis*-10-Heptadecanoic acid, *n*–7)	C17H32O2	267.2	267.2	10.25	Negative	157	5	12	10	12
FFA 18:0 (Stearic acid)	C18H36O2	283.05	283.05	14.45	Negative	158	5	18	10	15
FFA 18:1 (Oleic acid, *n*–9 or *cis*-Vaccenic acid, *n*–7)	C18H34O2	280.9	280.9	11.86	Negative	159	5	12	10	12
FFA 18:1 (Elaidic acid, *n*–9 or *trans*-Vaccenic acid, *n*–7)	C18H34O2	280.9	280.9	12.40	Negative	159	5	19	10	19
FFA 18:2 (Linoleic acid, *n*–6)	C18H32O2	278.95	278.95	9.76	Negative	160	5	21	10	21
FFA 18:3 (α-Linolenic acid, *n*–3 or γ-Linolenic acid, *n*–6)	C18H30O2	276.9	276.9	8.31	Negative	161	5	18	10	15
FFA 18:4 (Stearidonic acid, *n*–3)	C18H28O2	275.2	275.2	6.71	Negative	162	5	18	10	15
FFA 19:0 (Tuberculostearic acid)	C19H38O2	296.9	296.9	16.20	Negative	163	5	14	10	14
FFA 20:0 (Arachidic acid)	C20H40O2	311	311	17.52	Negative	164	5	14	10	14
FFA 20:1 (*cis*-11-Eicosenoic acid, *n*–9)	C20H38O2	309.3	309.3	15.14	Negative	165	5	14	10	14
FFA 20:2 (*cis*-11-14-Eicosadienoic acid, *n*–6)	C20H36O2	307.3	307.3	13.01	Negative	166	5	10	10	10
FFA 20:3 (Dihomo-γ-linolenic acid, *n*–6 or Mead acid, *n*–9)	C20H34O2	305.05	305.05	11.20	Negative	167	5	18	10	15
FFA 20:4 (Arachidonic acid, *n*–6)	C20H32O2	303.1	303.1	9.96	Negative	168	5	18	10	15
FFA 20:5 (Eicosapentaenoic acid, *n*–3)	C20H30O2	300.9	300.9	8.27	Negative	169	5	22	10	22
FFA 21:0 (Heneicosanoic acid)	C21H42O2	325.3	325.3	18.85	Negative	170	5	18	10	15
FFA 22:0 (Behenic acid)	C22H44O2	339.3	339.3	20.40	Negative	171	5	18	10	15
FFA 22:1 (Erucic acid, *n*–9)	C22H42O2	337	337	17.94	Negative	172	5	18	10	15
FFA 22:4 (Docosatetraenoic acid, *n*–6)	C22H36O2	331.3	331.3	12.75	Negative	173	5	18	10	15
FFA 22:5 (Docosapentaenoic acid, *n*–6)	C22H34O2	329.2	329.2	11.52	Negative	174	5	16	10	16
FFA 22:6 (Docosahexaenoic acid, *n*–3)	C22H32O2	326.95	326.95	9.84	Negative	175	5	18	10	15
FFA 23:0 (Tricosanoic acid)	C23H46O2	353.3	353.3	21.20	Negative	176	5	18	10	15
FFA 24:0 (Lignoceric acid)	C24H48O2	367.4	367.4	22.31	Negative	177	5	18	10	15
FFA 24:1 (Nervonic acid, *n*–9)	C24H46O2	365.3	365.3	20.35	Negative	178	5	18	10	15
FFA 25:0 (Pentacosanoic acid)	C25H50O2	381.4	381.4	22.97	Negative	179	5	18	10	15
FFA 26:0 (Cerotic acid)	C26H52O2	395.4	395.4	24.27	Negative	180	5	18	10	15
FFA 27:0 (Heptacosanoic acid)	C27H54O2	409.4	409.4	25.18	Negative	181	5	18	10	15
FFA 28:0 (Montanic acid)	C28H56O2	423.3	423.3	26.19	Negative	182	5	19	10	19
Cholic acid; CA (Cholic acid)	C24H40O5	407.1	407.1	2.47	Negative	183	5	29	10	29
Cholic acid; CDCA (Chenodeoxycholic acid)	C24H40O4	391.3	391.3	4.02	Negative	184	5	18	10	18
Cholic acid; UDGA (Ursodeoxycholic acid)	C24H40O4	391.3	391.3	2.12	Negative	184	5	18	10	18
Cholic acid; GCA (Glycocholic acid)	C26H43NO6	464.5	464.5	2.00	Negative	185	5	12	10	12
Cholic acid; GCDCA (Glycochenodeoxycholate)	C26H43NO5	448.1	448.1	2.81	Negative	186	5	30	10	30
Cholic acid; GDCA (Glycodeoxycholate)	C26H43NO5	448.1	448.1	3.05	Negative	186	5	30	10	30
Cholic acid; LCA (Lithocholic acid)	C24H40O4	375.1	375.1	5.64	Negative	187	5	26	10	26
Cholic acid; TCA (Taurocholate)	C26H45NO7S	514.3	514.3	1.94	Negative	188	5	24	10	24
Cholic acid; TCDCA (Taurochenodeoxycholate)	C26H45NO6S	498.5	498.5	2.59	Negative	189	5	23	10	23

Ether-linked isobaric species of plasmanyl (e) and plasmenyl (p) analogs of glycerophospholipids. Acyl positions of glycerolipids (sn-1 and sn-2).

Double bond positions (n).

IS, Internal Standard.

Q1 and Q3 pre-bias are the characteristic parameters of Shimadzu instrument.

**Figure 2:**
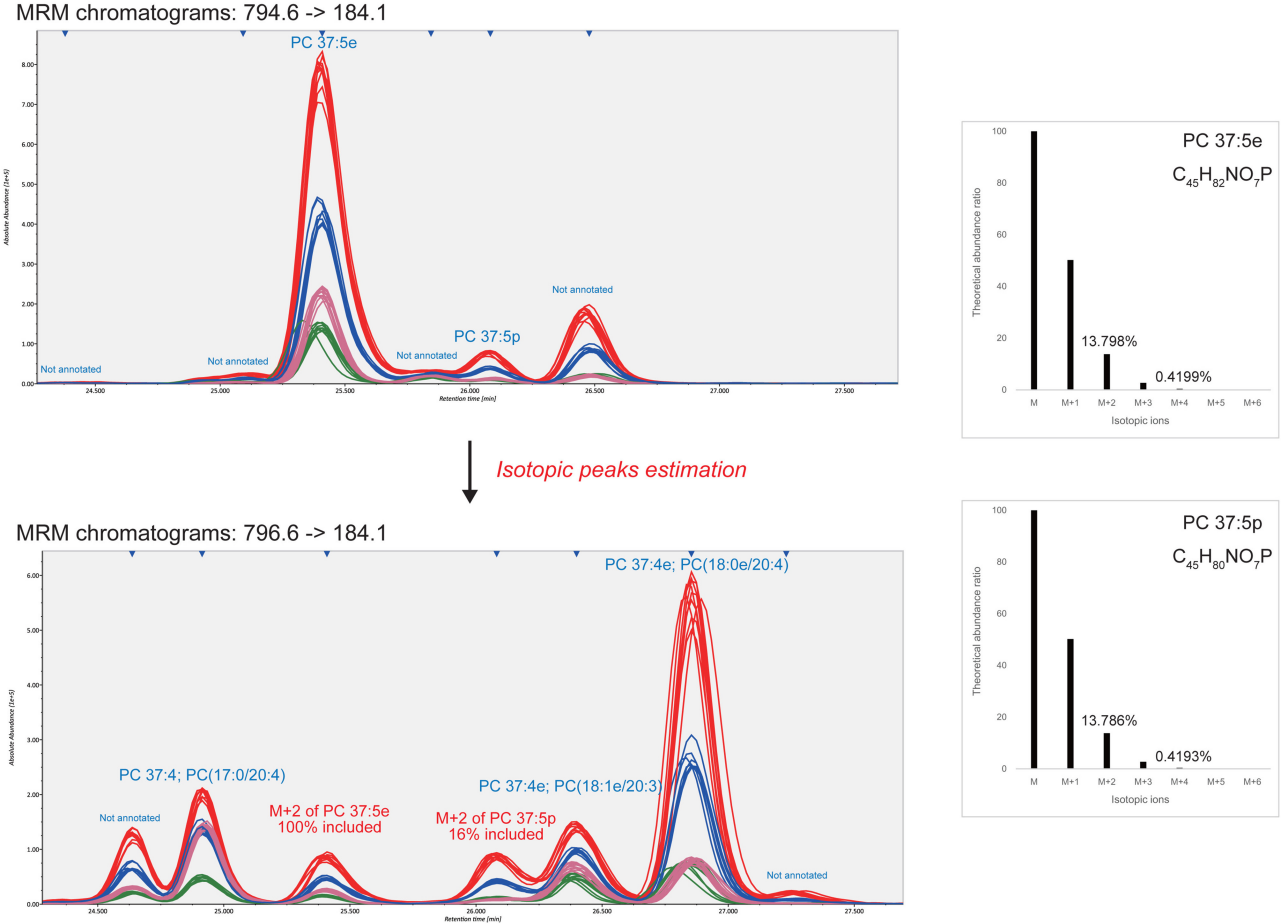
**Identification and quantification results from isotopic peak estimations**. This example demonstrates how the isotopic peaks affect compound identifications and quantifications. The 13.798 and 13.786% of the monoisotopic peak abundances of PC 37:5e and PC 37:5p are theoretically monitored at the M+2 MRM transition. As shown in the bottom-left figure, two detected peaks eluted at 25.4 and 26.1 min were derived from 100 and 16% of the respective monoisotopic ions. This result shows that the isotopic peaks should be estimated for compound identifications and quantifications.

The advantage of our strategy is the extensive use of isotopic peaks to reduce mis-identification and over-quantification. For example, in the 796.6–184.1 MRM chromatogram (Figure 2) the isotopic ion M+2 of highly abundant O-alkyl PC 37:5e (MW 794.6) was monitored at 25.4 min. Likewise, the isotopic ion M+2 of O-alkenyl phosphatidylcholine PC 37:5p (MW794.6) existed at 26.1 min but the isotope contributed only 16% of the total abundance, leaving 84% for another unidentified isomer. Therefore, to distinguish the isomer- and isotopic-peaks objectively, the MRM transitions for all patterns of different degrees of unsaturation (a ladder of 2 *m/z* decrements) must be monitored. The 796.6–184.1 MRM chromatogram contained six isomeric peaks including two unannotated peaks (24.6 and 27.3 min) and one isotopic peak from MW 794.6 (25.4 min). In our serum dataset, 71 significant peaks were dismissed as pure isotopic peaks of higher abundances.

**Figure 3:**
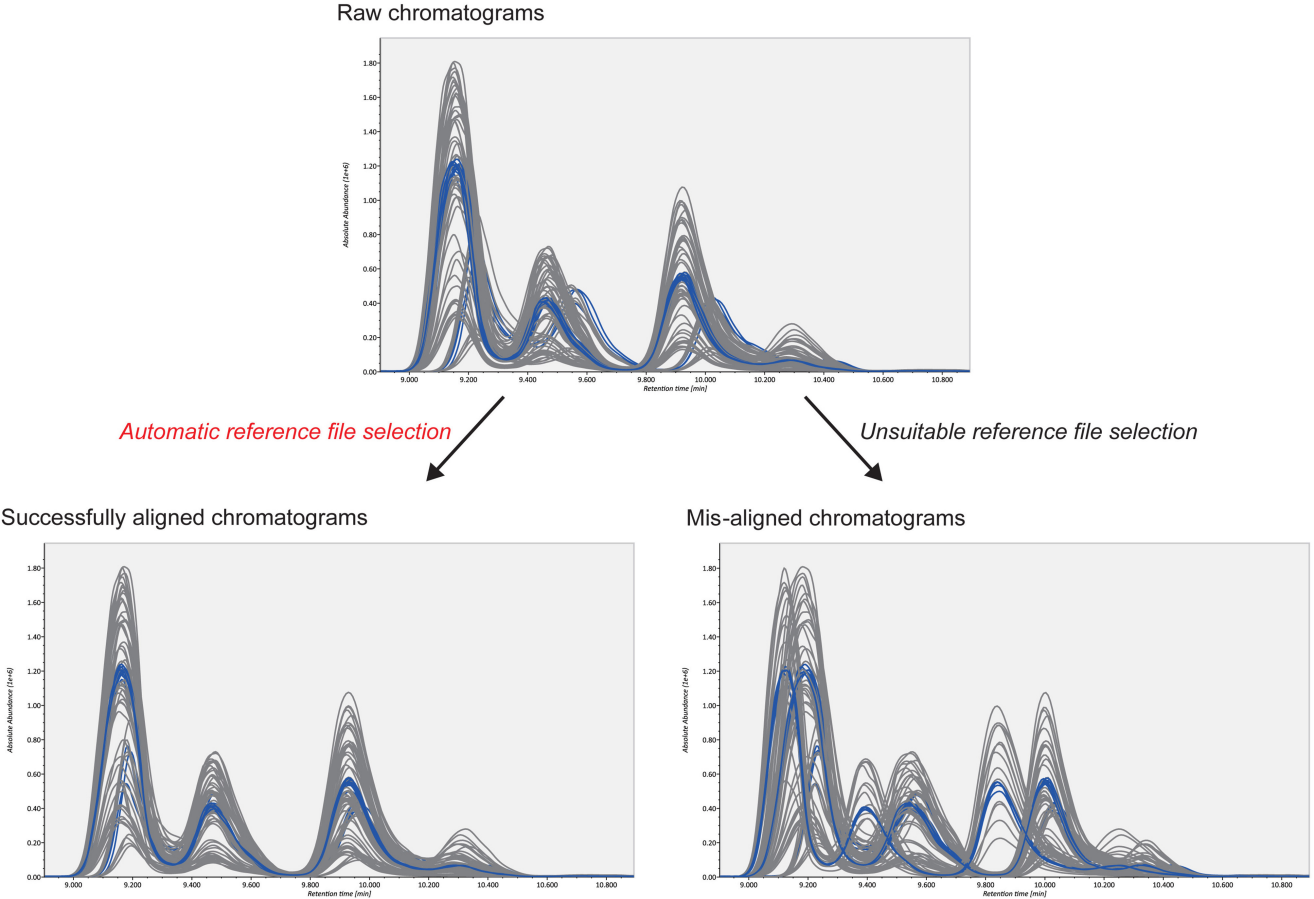
**Alignment results from two different reference files**. The upper figure shows raw chromatograms; the blue and gray lines describe pooled QCs and biological samples, respectively. As shown in the bottom figures, alignment accuracy is considerably affected by the selected reference file. The automatic reference selection method in combination with pooled QC datasets robustly picks the suitable reference file. This contributes to better-aligned results.

The second advantage is the smart selection of a QC reference for COW-based chromatographic alignment. The accurate alignment of MRM chromatograms is necessary for accurate lipid quantification. For reverse-phase LC methods, two user-defined parameters in the COW algorithm, “*segment size*” and “*warp slack*,” can be set as the peak width (0.5 min in our study) and as “1 or 2,” respectively. This leaves the selection of the reference chromatogram as the only critical parameter in the algorithm (Figure 3). The reference chromatogram should be positioned at the center of all chromatograms to be aligned. Moreover, the higher the chromatographic similarity, the better is the alignment. Therefore, we created pooled QC datasets as the average of all biological samples and chose one representative QC datum whose chromatogram was closest to the midpoint of all chromatograms (chromatographic centroid). The automatic selection picked the suitable reference and corrected the retention time drifts efficiently in our demonstration. Our example data sets are downloadable at http://prime.psc.riken.jp/.

**Figure 4:**
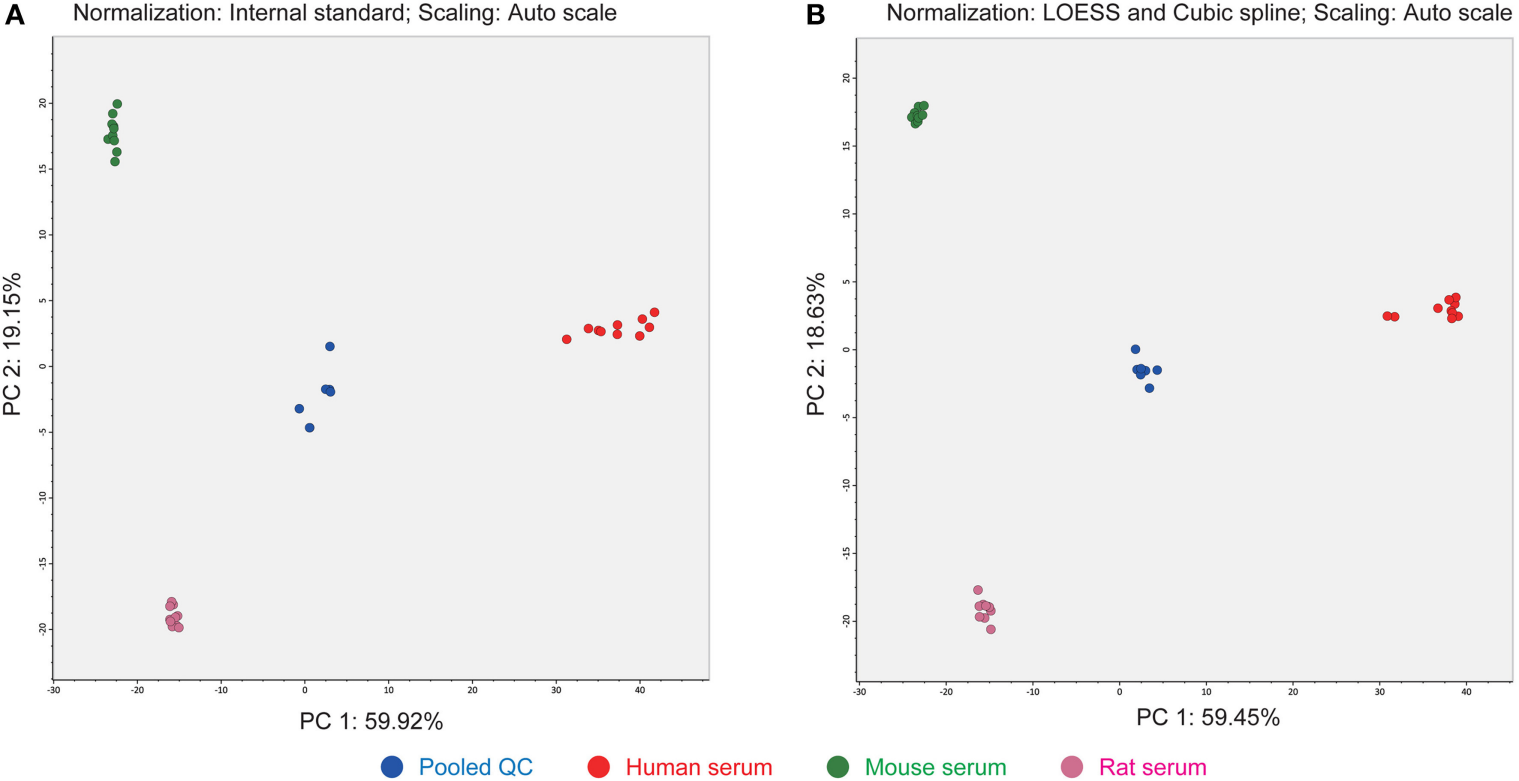
**Differences in PCA score plots of internal standard- and LOESS/cubic spline-based normalizations**. **(A)** The metabolite signals were normalized by one internal standard PC (12:0/12:0). **(B)** The metabolite signals were corrected by LOESS and cubic spline in combination with the abundances of pooled QC datasets that were analyzed at every five biological samples. This result shows that the clusters of each serum class were much improved with LOESS and cubic spline normalization.

The third advantage of our strategy is the use of LOESS and cubic spline normalization to reduce MS signal drifts in an analytical batch (Dunn et al., 2011). The LOESS smoother is first applied to the pooled QC datasets in the order of injection. Then the abundances of each metabolite in the biological samples are corrected by cubic spline interpolated from the abundances in the smoothed QC data. The score plot of principal component analysis (PCA) indicated a better decrease in the deviation of clustering results than when alternative normalization using an internal standard, PC (12:0/12:0), was applied (Figure 4). There is no consensus for normalization methods in the LIPID MAPS consortium (Ivanova et al., 2007) and MRM-DIFF provides for the application of both methods for abundance normalization. Most lipid compounds are commonly detected among human-, mouse-, and rat-sera. It seems that anabolic or cleavage enzymes such as fatty acid synthase and lipase are conserved at least for the major lipids identified in our study. In addition, the fatty acid varieties of human serum conjugated in glycerophospholipids were richer than in the other two species in which many PE species were not detected (Supplementary File 1).

## Discussion

We demonstrated a different analysis with the systematic strategy for MRM-based lipid profiling. Our MRM-DIFF software program semi-automatically performs lipid identification and quantification of large scale MRM datasets. It also considers isotopic peaks to reduce false-positives and mis-quantifications. While pooled QC data are not always necessary, they help to find system parameters and to correct MS signal intensities for a better performance.

The advantage of pattern recognition from overlays is that many candidates can be detected as unknown or identified compounds (Ma et al., 2008; Vallejo et al., 2009). In this study, 677 unknown compounds were reliably detected in serum samples from three species. Such metabolites can be validated one-by-one by authentic standards or by high-resolution MS/MS platforms with structure prediction. In addition, the retention time of lipids in reverse phase LC methods can be theoretically estimated by the quantitative structure-retention relationship (QSRR) (Kaliszan, 2007; Audain et al., 2014; Cao et al., 2014), and its accuracy is improving. The combination of MRM-DIFF and QSRR methods may ease the tedious task of molecular identification in the future. Indeed, the sensitivity and selectivity of triple quadrupole MS for lipids far exceeded those of TOF- or Orbitrap-MS with respect to the identified lipids (data not shown). The reliability of quantification can be therefore improved by the higher signal to noise ratio. On the other hand, high-resolution MS systems have the advantage in its qualitative aspect: the O-alkyl or O-alkenyl derivatives can be distinguished from standard acyl derivatives by accurate masses.

We introduced MRM-DIFF as a differential analysis tool for large-scale MRM assays of up to 200 datasets. However, the classical “widely targeted” approach remains important for analyzing lipids by MRM assays. We developed another software program, MRMPROBS, to support the analysis of MRM assays by setting two or three transitions for each molecular target (Tsugawa et al., 2014). Either of the two software programs, MRM-DIFF or MRMPROBS, can be chosen depending on the research needs.

## Materials and methods

### Reagents and chemicals

Human-, mouse-, and rat-sera were purchased from Sigma-Aldrich Co. (St. Louis, MO, USA), authentic standard compounds from Sigma-Aldrich and Avanti Polar Lipids, Inc. (Alabaster, AL, USA), and ammonium acetate from Sigma-Aldrich. LC/MS grade distilled water and LC/MS grade methanol were purchased from Wako Pure Chemical Industries Ltd. (Osaka, Japan).

### Sample preparation

Lipid extraction from the sera was as described previously (Yamada et al., 2013) with minor modifications. Briefly, 10 μL of serum were mixed into 90 μL of methanol containing 10 μL of PC 12:0/12:0 (0.5 μg mL^−1^) as the internal standard. After vortexing at the maximum setting for 1 min, the samples were placed on ice for 10 min. The extracts were then centrifuged at 16,000 × g for 5 min at 4°C and the resulting supernatant (60 μL) was recovered. QC sample (150 μL) was prepared by mixing equal amounts (10 μL each) of human-, mouse-, and rat-serum extract (*n* = 5).

### LC/MS/MS conditions

The LC/MS/MS system was comprised of a Shimadzu Nexera ultra-high-performance liquid chromatograph and a Shimadzu LCMS-8040 triple quadrupole mass spectrometer equipped with an ESI ion source (Shimadzu Co., Kyoto, Japan). The conditions for LC/MS/MS analysis were: column, InertSustain (2.1 × 150 mm; particle size, 3 μm; GL Sciences Inc., Tokyo, Japan); column temperature, 40°C; mobile phase, 20 mM ammonium acetate in water (A) or methanol (B); flow rate, 0.35 mL min^−1^; gradient curve, 75% B at 0 min, 99% B at 22 min, 99% B at 39 min, 75% B at 39.1 min, and 75% B at 45 min; injection volume, 2 μL; mass analysis mode, both positive and negative ion mode with a polarity switching time of 15 ms; electrospray voltage, 4.5 kV for positive- and −3.5 kV for negative-ion mode; nebulizer gas flow, 3.0 L min^−1^; drying gas flow, 15.0 L min^−1^; desolvation temperature, 250°C; heat block temperature, 400°C; and detector voltage, 1.62 kV. The MRM mode with a dwell time of 5 ms per channel was used. Other optimized MRM parameters for each lipid and its related metabolite are shown in Table 1. One pooled QC sample was repeatedly analyzed at each fifth sample injection in this study.

### Identification of lipids

Major glycerophospholipids such as phosphatidylcholine (PC), phosphatidylethanolamine (PE), lysophosphatidylcholine (lysoPC), and lysophosphatidylethanolamine (lysoPE) manifest a wide variety of structures with a hydrophilic head group and/or hydrophobic fatty acid tails that include isobaric and isomeric lipid molecular species in the serum. The workflow for the identification of the glycerophospholipids in the human serum using the LC/MS/MS system is shown in Supplementary Figure 1. In short, the selection of glycerophospholipid candidates was based on previously reported information (Quehenberger et al., 2010) and our experimental findings with LC/MS/MS based virtual MRM screening (Supplementary Tables 1, 2). Identification of the PC, PE, lysoPC, and lysoPE molecular species on a triple quadrupole mass spectrometer requires the detection of specific fragment ions derived from both the head group (phosphocholine, *m/z* 184.1 or phosphatidylethanolamine, *m/z* M-140.0) in the positive-ion mode and the fatty acid (FA) moieties in the negative-ion mode. Therefore, after the head group moieties of targeted glycerophospholipids were confirmed by LC/MS/MS with MRM in the positive-ion mode, the FA moieties of targeted glycerophospholipids were identified by product-ion scanning on the triple quadrupole mass spectrometer in the negative-ion mode. In some cases, more than two different molecular species (structural isomers) were detected within the same *m/z* peaks, which was difficult to separate by our LC condition. Additionally, to distinguish plasmanyl (e) and plasmenyl (p) analogs of glycerophospholipids, the acid hydrolysis analysis was performed according to the previous report (Taguchi and Ishikawa, 2010). Structural characterization of two lysoglycerophospholipid regioisomers such as 2-acyl-1-lysophospholipids (e.g., lysoPC 14:0 *sn*-2) and 1-acyl-2-lysophospholipids (e.g., lysoPC 14:0 *sn*-1) was also performed using C18-based reverse phase column chromatography (Okudaira et al., 2014). Other lipids such as free fatty acid (FFA), acylcarnitine (AC), cholesterol, and cholic acid (CA) were identified on the basis of the manual curation with their authentic standards. Finally, the compound name, the molecular formula, the MRM transition, and the RT of 284 lipids molecular species were stored in the user-defined lipids library (Table 1).

### MRM-DIFF software and data processing parameters

The MRM-DIFF program, the demonstration data set, and the tutorial are downloadable at the “Standalone software” section of PRIMe (Platform for RIKEN Metabolomics, http://prime.psc.riken.jp/) database website. MRM-DIFF can import two data formats: “Analysis Base File” (ABF) format converted by our file converter (Tsugawa et al., 2014) and the common mzML format converted by the ProteoWizard *MSConvert* software (Kessner et al., 2008). The ABF file converter is freely available at http://www.reifycs.com/english/AbfConverter/.

MRM-DIFF is available in Windows OS (.NET Framework 4.0 or later; RAM: 4.0 GB or more). Its source code was written in the C# language with the Windows Presentation Foundation (WPF) to develop the graphical user interface.

In this study, Shimadzu LCD files were converted to ABF format by our converter. The reference library for lipid identification was prepared from the above identification criteria and the library is downloadable at our RIKEN PRIMe website. After importing ABF files in the MRM-DIFF program, data processing was performed with the following parameters: smoothing method, linear weighted moving average; smoothing level, 2 scan; minimum peak width, 5 scan; minimum peak height, 100 amplitude; retention time tolerance, 0.2 min; minimum posterior, 70%; column type, ODS; segment size, 0.5 min; slack parameter, 1 scan; border limit, constant. The other details for the MRM-DIFF operation were described in the **MRM-DIFF tutorial** (http://prime.psc.riken.jp/).

### Theory

This section describes the mathematical methods implemented in the MRM-DIFF software program. MRM-DIFF accepts two data formats: the mzML data format converted via ProteoWizard and the ABF data format converted via our file converter program (Tsugawa et al., 2014). After importing all data files, the reference file is automatically selected by means of pooled QC datasets for each MRM transition as in the equation.

$$Chromatographic\;centroid=\frac{\begin{array}{l} \sum (abundance\left(n\right)*\\ \;retention\;time(n)) \end{array}}{\sum abundance(n)}$$

where *n* indicates the scan number of the chromatogram data points. This equation calculates the “*gravity*” of each chromatogram. MRM-DIFF selects a reference file whose value is closest to the midpoint between the minimum and the maximum of pooled QCs gravities.

We implemented COW (Nielsen et al., 1998) in the MRM-DIFF program as the non-linear alignment algorithm. Three parameters are required for chromatogram alignments, the *segment size*, *warp slack*, and a *targeted reference chromatogram*. The most important chore, selection of the reference chromatogram, is performed with the chromatographic centroid algorithm. Based on our experience, the segment size and warp slack parameters should be set to “peak widths (min)” and “1 or 2,” respectively, as long as reverse phase LC methods are used for lipid profiling. We also looked for suitable parameters for hydrophilic interaction chromatography (HILIC)- and pentafluorophenylpropyl (PFPP) columns; the recommended parameters are described in the **MRM-DIFF tutorial** (http://prime.psc.riken.jp/).

The peak detection algorithm is performed in the MRM-DIFF program. The principle underlying the peak detection method for pattern recognition is: (1) The peak detection method is applied to one representative chromatogram. The peak detection algorithm is the same as in our previous report (Tsugawa et al., 2014). (2) Peak detections for other chromatograms are based on a representative chromatogram; we call the “*data dependent peak detection method.*” (3) In this method, the local maximum within the left- and right edges of the representative peak is recognized as the peak top in the other chromatograms. (4) The left- and right edge of other chromatograms now corresponds with the representative peak in the MRM-DIFF program. Local minimum search from the assigned peak top is an alternative option. We highly recommend the peak height as the quantification value for detected peaks.

In the MRM-DIFF program, compound identification is based on retention time accuracy:

$$Retention\;time\;accuracy=\exp\;\left\{-0.5\times {\left(\frac{RT_{act.}-RT_{lib.}}{\delta }\right)}^{2}\right\}$$

*RT_act._* and *RT_lib._* indicate the measured- and reference retention time, respectively, and the σ value is the user-defined search tolerance. The value range is from 0 (non-consistency) to 1 (confidential); 0.7 is the default threshold for compound identifications. The criteria for isotopic ion estimation are: (1) isotopic ions up to M+6 are estimated. (2) MRM transitions including the same product ion are examined. (3) The abundance peaks higher than the monoisotopic ion are not recognized as the isotopic peaks. (4) Peak top differences within 1 s from the peak top of monoisotopic ions are recognized as the isotopic peaks.

In addition, the isotopic peak abundances are calculated by the theoretical isotopic ratio of the molecular formula with the following method [Tsugawa et al. (2014). MS-DIAL: Data Independent MS/MS Deconvolution for Comprehensive Metabolome Analysis, *submitted*]. For example, the isotopic ratio for C_42_H_82_NO_8_P, i.e., PC(16:0/18:1), is represented as the coefficient values of expanded elements of the following equation.

$$\begin{array}{l} {{(}^{12}\text{C}{+}^{13}\text{C})}^{42}{{(}^{1}\text{H}{+}^{2}\text{H})}^{82}{{(}^{14}\text{N}{+}^{15}\text{N})}^{1}{{(}^{16}\text{O}{+}^{17}\text{O}{+}^{18}\text{O})}^{8}{{(}^{31}\text{P})}^{1}=\\ {[}^{12}{\text{C}}_{42}^{1}{\text{H}}_{82}{}^{14}{\text{N}}_{1}{}^{16}{\text{O}}_{8}{}^{31}{\text{P}}_{1}]{\left(1+\frac{{}^{13}\text{C}}{{}^{12}\text{C}}\right)}^{42}{\left(1+\frac{{}^{2}\text{H}}{{}^{1}\text{H}}\right)}^{82}{\left(1+\frac{{}^{15}\text{N}}{{}^{14}\text{N}}\right)}^{1}\\ \;{\left(1+\frac{{}^{17}\text{O}}{{}^{16}\text{O}}+\frac{{}^{18}\text{O}}{{}^{16}\text{O}}\right)}^{8} \end{array}$$

The letter such as ^12^C indicates the natural abundance of each element. The above contents except for the mono-isotopic mass ^12^C^1^_42_H^14^_82_N^16^O^31^_8_P are expanded. The coefficient value of each expanded term represents the relative isotope abundances with respect to the mono-isotopic ion. Since only nominal masses are output from the triple quadrupole MS system, the theoretical abundances are binned into nominal values.

Finally, the LOESS/cubic spline method was implemented as described in our previous report (Tsugawa et al., 2014). Note that the MRM-DIFF program does not require pooled QC datasets. The QC datasets can be alternated with other files given the above mathematical details. However, the LOESS/cubic spline normalization method cannot be applied.

### Conflict of interest statement

The authors declare that the research was conducted in the absence of any commercial or financial relationships that could be construed as a potential conflict of interest.
