# Supplementary material for: MRM-DIFF: data processing strategy for differential analysis in large scale MRM-based lipidomics studies
Source: Front Genet. 2015 Jan 30;5:471. doi: 10.3389/fgene.2014.00471 (PMC4311682; doi:10.3389/fgene.2014.00471)
Supplement: Supplementary file 4 [file Image1.PDF]

1. Selection of glycerophospholipid candidates (i.e., lysoPC, PC, lysoPE, and PE) based on previously reported information (Quehenberger et al., 2010) and our experimental findings with LC/MS/MS based virtual MRM screening in the positive-ion mode (Tables S1 and S2).

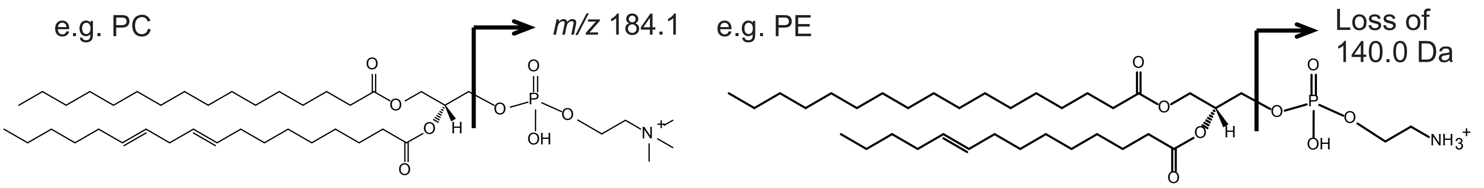

2. Peak detection and the determination of the precursor ion of individual glycerophospholipids (i.e., discrimination between monoisotopic peak and isotopic peaks) using MRM-DIFF program.

3. Determination of the FAs moieties of targeted glycerophospholipids by product-ion scanning on the triple quadrupole mass spectrometer in the negative-ion mode.

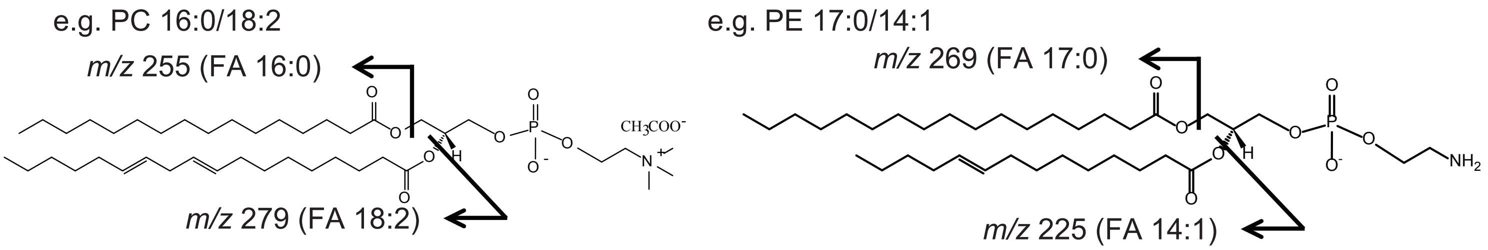

4. Discrimination between plasmanyl (e) and plasmenyl (p) analogues of glycerophospholipids by acid hydrolysis analysis (Taguchi and Ishikawa, 2010).
The peaks of plasmenyl (p) glycerophospholipids were eliminated by addition of 1 M HCl.
On the other hand, the peaks of plasmanyl (e) species were not eliminated by addition of 1 M HCl.

5. Structural characterization of two lysoglycerophospholipid regioisomers such as lysoPC 14:0 *sn*-2 and lysoPC 14:0 *sn*-1 based on the elution order of C18-based reverse phase column chromatography (Okudaira et al., 2014).

6. Storage of the compound name, the molecular formula, the MRM transition, and the RT to the user-defined lipids library (Table 1).
